# The *rpl23* gene and pseudogene are hotspots of illegitimate recombination in barley chloroplast mutator seedlings

**DOI:** 10.1038/s41598-019-46321-6

**Published:** 2019-07-10

**Authors:** F. Lencina, A. M. Landau, M. E. Petterson, M. G. Pacheco, K. Kobayashi, A. R. Prina

**Affiliations:** 1 Instituto de Genética “Ewald A. Favret”, CICVyA (Centro de Investigación en Ciencias Veterinarias y Agronómicas), INTA (Instituto Nacional de Tecnología Agropecuaria), Nicolás Repetto y de los Reseros s/n (1686), Hurlingham, Buenos Aires Argentina; 2 Laboratorio de Agrobiotecnología, Grupo Biología Molecular Vegetal Aplicada, Instituto de Biodiversidad y Biología Experimental y Aplicada (IBBEA, CONICET-UBA), Departamento de Fisiología, Biología Molecular y Celular, Facultad de Ciencias Exactas y Naturales, UBA, Buenos Aires, Argentina

**Keywords:** Plant genetics, Plant molecular biology

## Abstract

Previously, through a TILLING (Targeting Induced Local Lesions in Genomes) approach applied on barley chloroplast mutator (*cpm*) seedlings a high frequency of polymorphisms in the *rpl23* gene was detected. All the polymorphisms corresponded to five differences already known to exist in nature between the *rpl23* gene located in the inverted repeats (IRs) and the *rpl23* pseudogene located in the large single copy region (LSC). In this investigation, polymorphisms in the *rpl23* gene were verified and besides, a similar situation was found for the pseudogene in *cpm* seedlings. On the other hand, no polymorphisms were found in any of those loci in 40 wild type barley seedlings. Those facts and the independent occurrence of polymorphisms in the gene and pseudogene in individual seedlings suggest that the detected polymorphisms initially arose from gene conversion between gene and pseudogene. Moreover, an additional recombination process involving small recombinant segments seems to occur between the two gene copies as a consequence of their location in the IRs. These and previous results support the hypothesis that the CPM protein is a component of the plastome mismatch repair (MMR) system, whose failure of the anti-recombination activity results in increased illegitimate recombination between the *rpl23* gene and pseudogene.

## Introduction

The plastid genome or plastome is considered more conserved evolutionarily in comparison to the nuclear genome in terms of its structural organization and gene content. However, comparative molecular analyses revealed complex patterns of mutational changes^1^ suggesting the existence of failures of DNA replication, recombination and/or repair (DNA-RRR) systems in the plastome^2^.

It is known that the stability of the plastome can be markedly affected by nuclear gene mutations, some of them also altering the genetic stability of the mitochondrion^3–5^. Investigations of these mutants could improve the limited knowledge about the mechanisms that maintain the integrity of plant organellar DNA.

**Figure 1:**
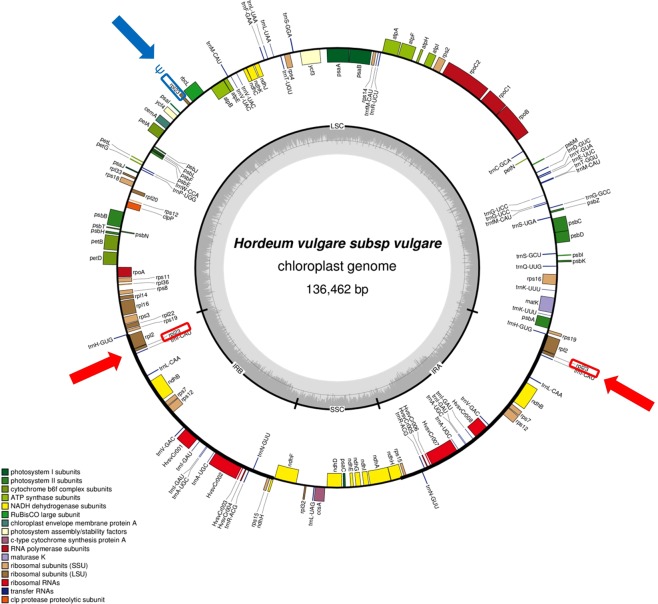
The chloroplast genome of barley. The *rpl23* gene copies located in the inverted repeats (IRs) are highlighted in red and pointed by red arrows. The *rpl23* pseudogene (Ψ) located in the large single copy region (LSC) is highlighted in blue and pointed by a blue arrow.

The barley chloroplast mutator (*cpm*) mutant is the first example in monocots that induces a wide spectrum of cytoplasmically inherited mutations^5–8^, which serve as a rich resource for the study of the mechanisms for the maintenance of plastome integrity. Previously, we proved that the plastome was the location of a number of mutations isolated from *cpm* plants^9–12^ and recently^13^, we extended the molecular analysis of the plastome in *cpm* seedlings through a TILLING (Targeting Induced Local Lesions in Genomes) approach. Following this strategy, we identified a wide range of plastome genes containing polymorphisms, consistent with our previous hypothesis about the involvement of the *Cpm* gene in maintaining plastome stability^6,14^. The spectrum of molecular changes observed in *cpm* seedlings mostly consisted of point mutations and small indels in microsatellite regions, which were explained as originating from at least 61 independent mutational events. However, in the case of the *rpl23* gene, we observed a peculiar pattern of variation, which consisted of combinations of one to several of the same five polymorphisms. In the grass family there are two copies of the *rpl23* gene that are located in the IRs, while there also exist a non-functional version, the *rpl23* pseudogene, located in the large single copy (LSC) region^15^ (see Fig. 1).

Coincidently, the five *rpl23* gene polymorphisms mentioned above correspond to the polymorphic variation already existing between the *rpl23* gene and pseudogene in nature, suggesting that they arose from recombination events between these two loci. In support of this hypothesis, Bowman *et al*.^15^ suggested that the *rpl23* pseudogene has been converted by the functional *rpl23* gene, and Morton and Clegg^16^ had provided additional support for a gene conversion model between the *rpl23* pseudogene and its functional counterpart in at least two lineages of the grass family.

In the present work, we performed a deeper analysis of the *rpl23* gene and included the *rpl23* pseudogene using the same DNA samples of *cpm* seedlings previously investigated by Landau *et al*.^13^. Moreover, both regions were also studied in two groups of control genotypes. One included several families derived from the parental line from which the *cpm* mutant was isolated^6^. The other group consisted of several barley genotypes representing a wide range of origins.

It was concluded that the nuclear *cpm* mutant induces not only a high rate of plastome point mutants, as previously reported^13^, but it also stimulates recombination between the *rpl23* gene and pseudogene, via gene conversion.

Our findings support previous hypotheses, which postulated that the *Cpm* gene is involved in plastome DNA repair^6^ as a component of the mismatch repair system^13^.

## Results

### Analysis of *rpl23* gene in polymorphic *cpm* seedlings

**Figure 2:**
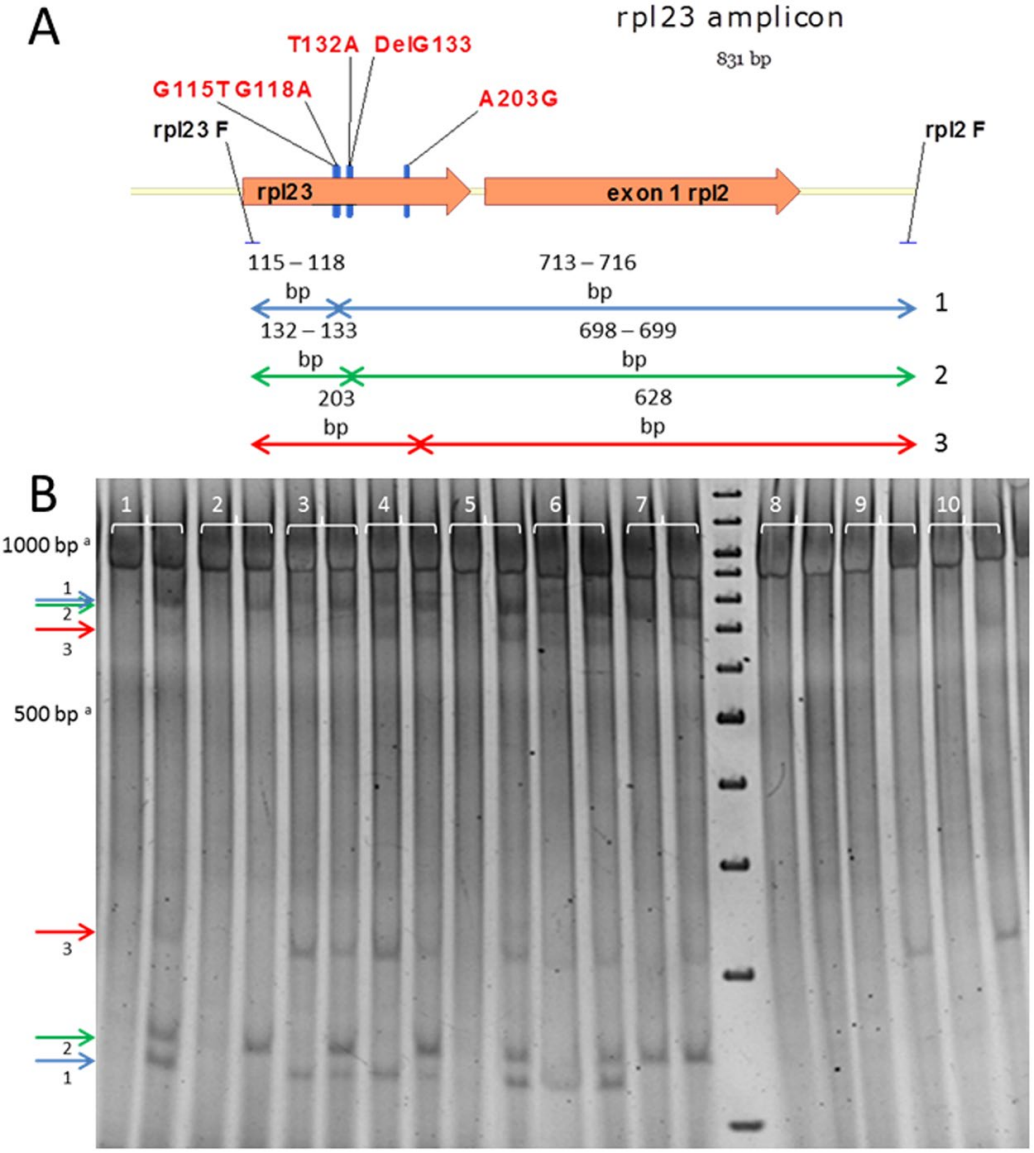
The *rpl23* amplicon. (**A**) Polymorphisms detected in the *rpl23* gene. (**B**) CJE digestions of *rpl23* amplicons corresponding to 10 *cpm* seedlings on a nondenaturing 3% polyacrylamide gel. The first and second lanes of each sample correspond to the digestion of the sample alone and mixed with wild-type DNA in a 1:1 ratio, respectively. The samples show different digestion patterns because they carry several combinations of polymorphisms: seedlings 1, 3, 4, 5 and 6 carry G115T, G118A, T132A, DelG133 and A203G; seedling 2 carries T132A and DelG133; seedling 7 carries T132A, DelG133 and A203G; seedling 8 does not carry any polymorphism and seedlings 9 and 10 carry A203G. ^a^The samples run slower than the molecular weight marker probably due to the components of the CJE digestion mixture.

A chloroplast TILLING (cpTILLING) analysis of the region corresponding to the *rpl2* amplicon in 304 *cpm* seedlings previously showed that 92 were polymorphic for the *rpl23* gene^13^. In the present study, the same *cpm* seedlings were further analyzed using the region corresponding to the *rpl23* amplicon (see Fig. 2A). The *rpl23* amplicon digestions showed three pairs of bands, as they did in the *rpl2* amplicon (Supplementary Fig. S1). However, the product bands of *rpl23* amplicon digestion were better resolved in the analytical gel (Fig. 2B) and, therefore, the different combinations of *rpl23* gene polymorphisms, *i*.*e*. genetic variants, could be more precisely evaluated. In fact, all of them were later confirmed by sequencing. These assessments showed that 30% (92/304) of the *cpm* seedlings were polymorphic for the *rpl23* gene.

**Figure 3:**
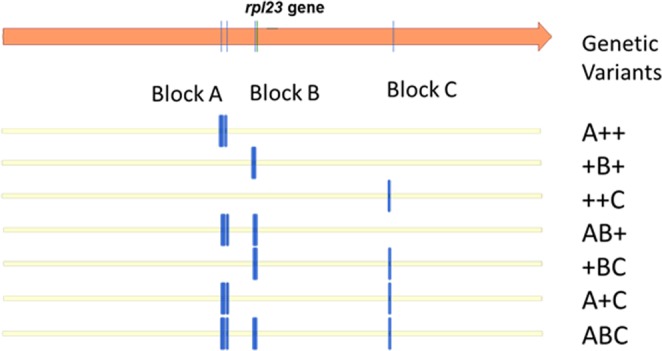
Representation of the potential genetic variants in the *rpl23* gene. A, B and C represent the polymorphic positions in the *cpm rpl23* gene (blocks A, B and C). +corresponds to the wild type sequence.

The sequencing of the *rpl23* amplicon confirmed that the different digestion patterns always represented combinations of five DNA molecular changes in the *rpl23* gene that correspond to the following mutations: 2 transitions (G118A: missense; A203G: missense), 2 transversions (G115T: missense; T132A: silent), and 1 nucleotide deletion (G at position 133: frameshift mutation generating a premature stop codon). As depicted in Fig. 3 and detailed in Table 1, these five changes were always observed in three groups or blocks named **A**, **B** or **C**, while the absence of molecular changes (corresponding to the wild type sequence) is represented by the symbol + in each of the blocks. Block **A** comprised two single base mutations (G115T and G118A), as did block **B** (T132A and DelG133), while block **C** consisted of only one molecular change (A203G). The different block combinations (**ABC**, **AB+**, etc.) can give seven potential genetic variants, in addition to the wild type sequence in all the three blocks (**+++**).

**Table 1:** Number of seedlings carrying different *rpl23* gene variants in the two groups of *cpm* families.

Genetic variants in the *rpl23* gene^a^								
	A++	+B+	++C	AB+	+BC	A + C	ABC^b^	+++^c^
**Group A**								
F_2_	0	4 (1)	25 (23)	4 (0)	6 (0)	0	12 (5)	108
M_2_	0	0	5 (5)	0	0	0	1 (0)	17
**Subtotal**	**0**	**4 (1)**	**30 (28)**	**4 (0)**	**6 (0)**	**0**	**13 (5)**	**125**
**Group B**								
F_2_-1	0	0	3 (2)	0	1 (0)	0	1 (0)	21
F_2_-2	0	0	1 (0)	0	3 (2)	0	0	12
F_2_-3	0	1 (0)	2 (0)	4 (0)	1 (0)	0	4 (0)	27
F_2_-4	1 (0)	5 (0)	5 (1)	0	0	0	3 (0)	27
**Subtotal**	**1 (0)**	**6 (0)**	**11 (3)**	**4 (0)**	**5 (2)**	**0**	**8 (0)**	**87**
**Total**	**1 (0)**	**10 (1)**	**41 (31)**	**8 (0)**	**11 (2)**	**0**	**21 (5)**	**212**

^a^The number of homoplastomic seedlings is shown in brackets. ^b^A, B and C represent the polymorphic positions in the *cpm rpl23* gene (blocks A, B and C). ^c^+represents the wild type sequences.

All potential combinations, with the exception of **A + C**, were observed for the *rpl23* gene (Table 1). The most abundant variant was ++**C**, which was observed in all the six families and in 41 out of the 92 *rpl23* gene polymorphic seedlings. It was followed by variant **ABC**, which was observed in five families and 21 seedlings.

### Screening and identification of *rpl23* pseudogene polymorphisms in *cpm* seedlings

**Figure 4:**
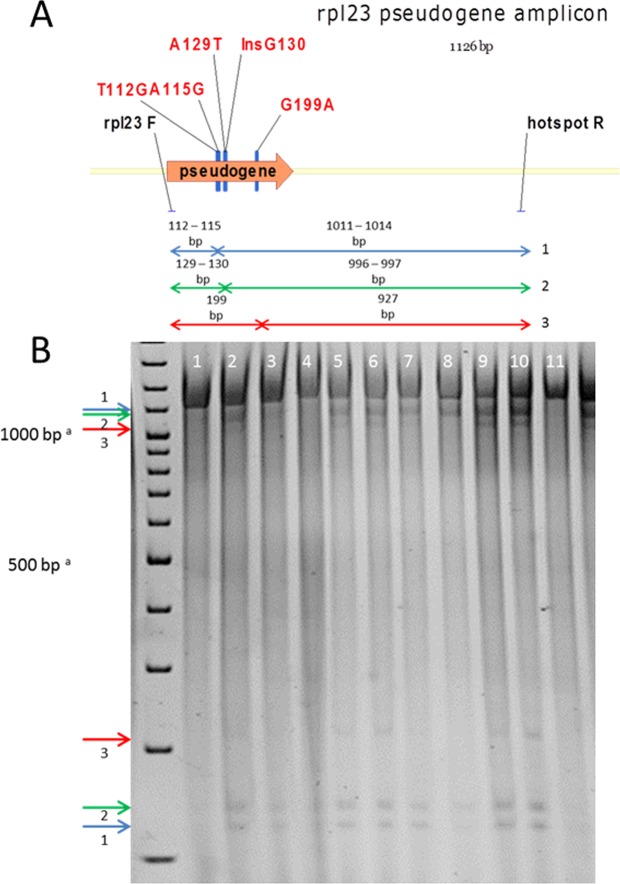
The *rpl23* pseudogene amplicon. (**A**) Polymorphisms detected in the *rpl23* pseudogene. (**B**) CJE digestions of *rpl23* pseudogene amplicons on a nondenaturing 3% polyacrylamide gel. The *cpm* samples show different digestion patterns because they carry several combinations of polymorphisms: seedlings 1, 4 and 11 do not carry any polymorphism; seedlings 2, 3 and 8 carry T112G, A115G, A129T and InsG130; seedlings 5, 6, 7, 9 and 10 carry T112G, A115G, A129T, InsG130 and G199A. ^a^The samples run slower than the molecular weight marker probably due to the components of the CJE digestion mixture.

All 304 *cpm* seedlings previously analyzed by Landau *et al*.^13^ to isolate the 92 *rpl23* gene polymorphic seedlings were tested in the present investigation for *rpl23* pseudogene polymorphisms, by using a similar strategy of CJE digestion of the *rpl23* pseudogene amplicon (see Fig. 4A). As observed for the *rpl23* gene, combinations of three pairs of bands were identified (Fig. 4B), whose sizes were similar to those of the *rpl23* gene.

**Figure 5:**
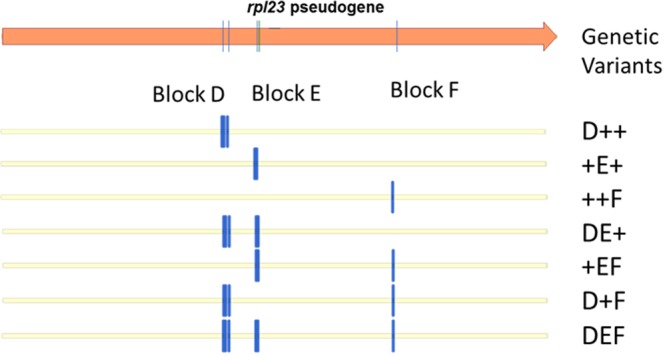
Representation of the potential genetic variants in the *rpl23* pseudogene. D, E and F represent the polymorphic positions in the *cpm rpl23* pseudogene (blocks D, E and F). +corresponds to the wild type sequence.

The sequencing of *rpl23* pseudogene amplicons giving differential digestion patterns revealed that all the genetic variants corresponded to combinations of the five molecular changes observed in *rpl23* pseudogene: two transitions (A115G and G199A), two transversions (T112G and A129T) and 1 nucleotide insertion (G at position 130). Moreover, as observed for the *rpl23* gene, the five mutations in the *rpl23* pseudogene appeared to be grouped in three blocks, which were named **D**, **E** and **F**. Block **D** contained two molecular changes (T112G and A115G), as did block **E** (A129T and InsG130), while block **F** contained only one molecular change (G199A) (see Fig. 4). Also similar to the *rpl23* gene, the potential combinations of these blocks would give seven genetic variants for the *rpl23* pseudogene besides the combination composed of the wild type pseudogene sequences in all three blocks +++ (Fig. 5).

The number of seedlings carrying the different *rpl23* pseudogene variants in each family is shown in Table 2. The *rpl23* pseudogene was polymorphic in 65% of the seedlings (197 out of 304). Although seedlings polymorphic for the *rpl23* pseudogene were approximately twice than those polymorphic for the *rpl23* gene, only three of the seven potential genetic variants were detected. Most of *rpl23* pseudogene polymorphic seedlings (128 out of 197) carried the **DEF** combination with changes in all three blocks. The variant **DE + **was observed in 66 out of 197 seedlings, while only three seedlings showed the variant **D + F**. This means that almost all *rpl23* pseudogene polymorphic seedlings (194 out of 197) carried only two of the potential combinations.

**Table 2:** Number of seedlings carrying different *rpl23* pseudogene variants in the two groups of *cpm* families.

Genetic variants in the *rpl23* pseudogene^a^								
	D++	+E+	++F	DE+	+EF	D + F	DEF^b^	+++^c^
**Group A**								
F_2_	0	0	0	37 (22)	0	3(2)	57 (43)	62
M_2_	0	0	0	10 (8)	0	0	6 (6)	7
**Subtotal**	**0**	**0**	**0**	**47 (30)**	**0**	**3 (2)**	**63 (49)**	**69**
**Group B**								
F_2_-1	0	0	0	7 (4)	0	0	10 (5)	9
F_2_-2	0	0	0	0	0	0	11 (4)	5
F_2_-3	0	0	0	7 (2)	0	0	19 (11)	13
F_2_-4	0	0	0	5 (4)	0	0	25 (12)	11
**Subtotal**	**0**	**0**	**0**	**19 (10)**	**0**	**0**	**65 (32)**	**38**
Total	**0**	**0**	**0**	**66 (40)**	**0**	**3 (2)**	**128 (81)**	**107**

^a^The number of homoplastomic seedlings is shown in brackets. ^b^D, E and F represent the polymorphic positions in the *cpm rpl23* pseudogene (blocks D, E and F). ^c^+represents the wild type sequences.

### Screening for polymorphisms in the *rpl23* gene and *rpl23* pseudogene in wild type control seedlings

No polymorphisms for the *rpl23* gene or the *rpl23* pseudogene were detected in 40 DNA samples coming from individual wild type seedlings of the two groups of accessions used as controls using CJE digestions (Supplementary Figs S2 and S3). The absence of polymorphisms was further corroborated by sequencing both amplicons in some randomly selected control seedlings.

### Determination of the homo- or hetero-plastomic state for variations in the *rpl23* gene and *rpl23* pseudogene

The homo- or hetero-plastomic state of the variants in the *rpl23* gene and the pseudogene was estimated through the observation of a single or double peak, respectively, in the electropherograms after the amplicons were sequenced. In addition, CJE digestions were also performed. As a criterion, the blocks were considered to be homoplastomic when the digestion products were only detected in DNA samples previously mixed with wild type DNA, while they were considered to be heteroplastomic when the digestion bands were observed in the DNA sample analyzed alone. Only the seedlings carrying all the blocks in homoplastomic state were considered homoplastomic.

Thirty nine out of the 92 *rpl23* gene polymorphic seedlings were determined as homoplastomic by the two methods mentioned above; most of them (31 out of 39) had the variant combination **++C**. The other variants determined as homoplastomic were: **ABC**, **+BC** and +**B+**, which were found in five, two and one seedling respectively.

In contrast to what was observed in the *rpl23* gene, homoplastomic seedlings were much more frequent in the pseudogene, but the genetic variants were fewer. In fact, the majority of the *rpl23* pseudogene polymorphic seedlings were homoplastomic (123 out of 197) being the proportion of seedlings in homo- or heteroplastomic state not homogeneous between gene and pseudogene (χ^2^ = 10.23; df = 1; p = 0.0014). Most of the *rpl23* pseudogene homoplastomic seedlings (81 out of 123) had the genetic variant **DEF**, 40 seedlings had the variant **DE+** and only two seedlings had **D + F**.

Regarding the proportion of homoplastomic seedlings with respect to the two groups of *cpm* families analyzed (see Materials and Methods), in Group A the gene was observed homoplastomic in 34 out of 57 seedlings (0.60) *versus* 5 out of 35 (0,14) in Group B. While in Group A, the pseudogene was observed homoplastomic in 81 out of 113 seedlings (0.72) *versus* 42 out of 84 (0.50) in Group B. That is to say, the proportion of homoplastomic seedlings was higher in Group A than in Group B for both loci, gene and pseudogene.

### Analysis of albino seedlings carrying polymorphisms in the *rpl23* gene

Among the 304 *cpm* seedlings, 20 had an albino phenotype, and twelve of these were polymorphic for the *rpl23* gene, with different genetic variants. Eight of these seedlings were homoplastomic: five had the **ABC** combination (see digestions of samples 1 and 5 in Fig. 2B), two had the +**BC** variant and only one had the +**B+** variant (see digestion of sample 2 in Fig. 2B and the electropherogram in Supplementary Fig. S4). The remaining four albino seedlings were heteroplastomic for blocks **A** and **C** but not for block **B** (see digestions of samples 3, 4 and 6 in Fig. 2). Moreover, it was observed that none of the *rpl23* polymorphic seedlings with a normal green phenotype was homoplastomic for block **B**.

Furthermore, we looked for *striata-albina* seedlings in one of the families that segregated the albino seedlings mentioned above. The *striata* seedlings allowed us to separately isolate samples from normal green or albino tissues of the same seedling (Supplementary Fig. S5). When sequencing the *rpl23* amplicon in samples isolated from three individual seedlings, those from albino tissues showed the presence of block **B** in homoplastomic state, while those from normal green tissues showed the wild type sequence of the *rpl23* gene or the sequence corresponding to block **B** in heteroplastomic state (combination of variants +++ and **+B+**).

All these data show that there is an association between the homoplastomic state of block **B** (DelG133) and albinism.

### Analysis of RbcL protein level in albino and normal green seedlings by Western blot

**Figure 6:**
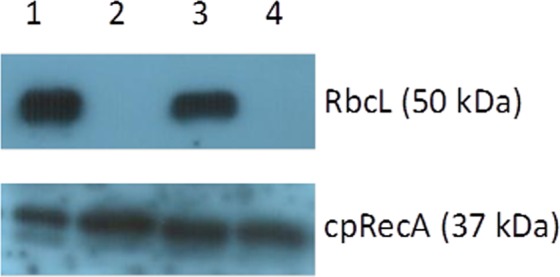
Immunoblot analysis of RbcL (upper) and cpRecA (lower) proteins in soluble protein extracts of albino and normal green seedlings from two different families. 20 µg of proteins were loaded per lane. Lanes 1 and 2: normal green and albino siblings, respectively. Lanes 3 and 4: normal green and albino siblings, respectively.

The deletion at position 133 of the *rpl23* gene causes a frameshift generating a premature stop codon and a truncated protein of only 47 amino acids (Supplementary Fig. S6), which is probably non-functional. This led us to investigate the level of the Rubisco large subunit (RbcL), to check if the chloroplast protein accumulation due to impairment of the chloroplast translation was affected in albino seedlings containing the DelG133 in homoplastomy. We also evaluated a nucleus-encoded chloroplast-targeted cpRecA protein, as a control. The presence or absence of these two proteins was determined by Western blots of progeny of *rpl23* polymorphic seedlings from two different families (see Materials and Methods) segregating albino and normal green seedlings. RbcL was missing in the albino seedlings of both families. In contrast, RbcL was detected in samples from green seedlings. On the other hand, the chloroplast targeted version of the nuclear-encoded protein cpRecA was present in both, albino and normal green seedlings (Fig. 6).

### Comparison of wild type sequences of the *rpl23* gene and pseudogene

**Figure 7:**
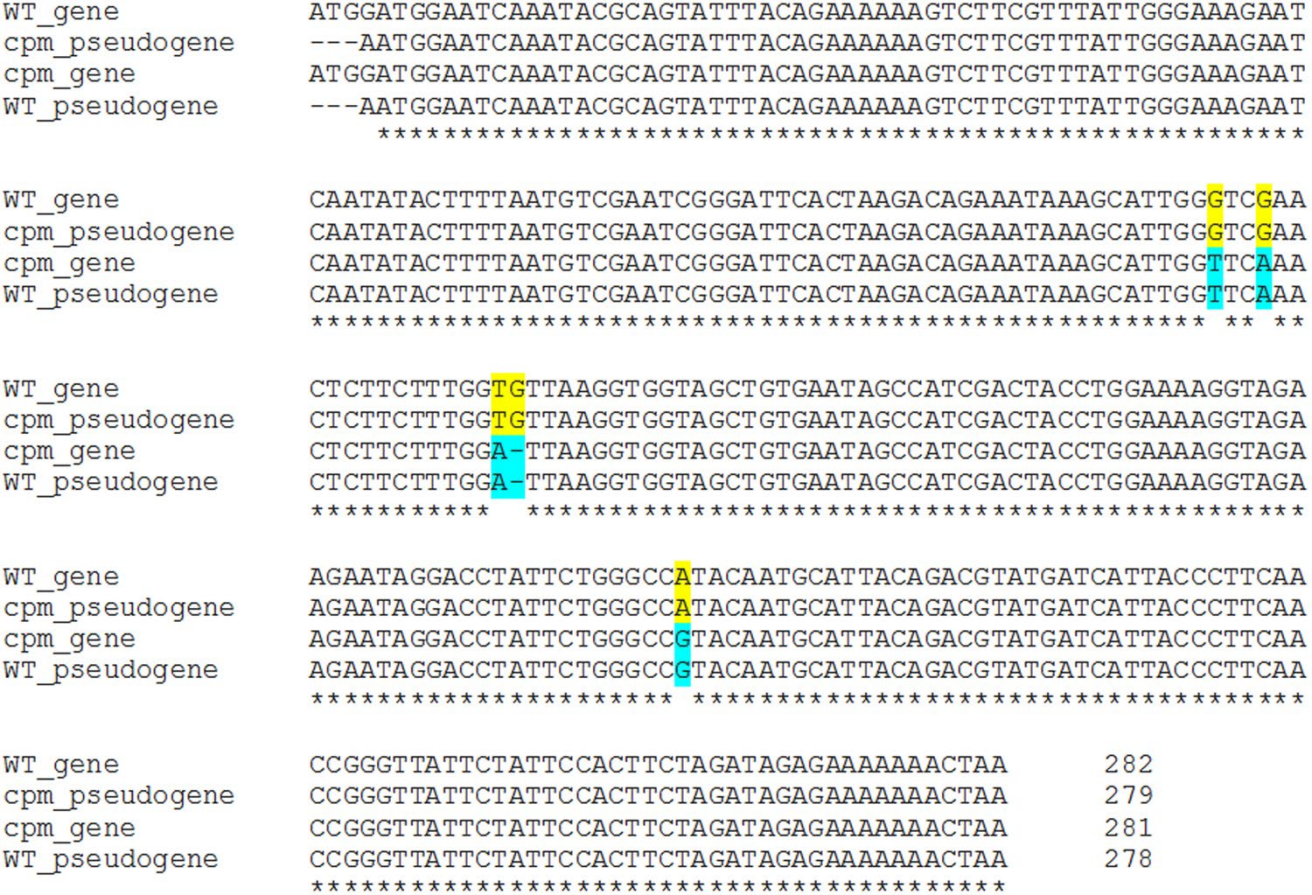
Alignment of *rpl23* gene and *rpl23* pseudogene sequences of wild type and polymorphic *cpm* versions.

The alignment of the barley wild type sequences of the *rpl23* gene and *rpl23* pseudogene showed five polymorphic positions in addition to the absence of ATG at the beginning of the *rpl23* pseudogene. Notably, these positions coincide exactly with blocks **A**, **B**, **C**, **D**, **E** and **F** mentioned above. The molecular changes identified in the *rpl23* gene of *cpm* seedlings match exactly with the corresponding sequence of the wild type pseudogene. Similarly, the molecular changes identified in the pseudogene of *cpm* seedlings match exactly with the corresponding sequence of the wild type gene of barley (Fig. 7).

In contrast to the high frequency of seedlings carrying the molecular changes corresponding to the genetic variants described above, notably no other polymorphisms were found in the *rpl23* gene or *rpl23* pseudogene loci. However, two different polymorphisms were found in both amplicons, but outside the gene or the pseudogene regions. One of them was a C302T substitution in the *rpl23* amplicon, which was found in only one seedling. The other one, which was also found in only one seedling, was a T856C substitution in the *rpl23* pseudogene amplicon.

### Coexistence of *rpl23* gene and *rpl23* pseudogene polymorphisms in individual seedlings

The coexistence of polymorphisms, *i*.*e*.*:* existence of polymorphisms in the *rpl23* gene and the *rpl23* pseudogene in individual seedlings, is presented in Table 3. Data were compiled per locus disregarding the variants or the heteroplastomic or homoplastomic state of the polymorphisms. The major proportion of seedlings had polymorphisms only in the pseudogene. Statistically, the occurrence of polymorphisms in the gene or the pseudogene are independent (χ^2^ = 0.47; df = 1; p = 0.49).

**Table 3:** Number of seedlings with polymorphic *rpl23* gene and/or *rpl23* pseudogene.

*rpl23* gene	*rpl23* pseudogene	Number of seedlings
polymorphic	polymorphic	57
polymorphic	wild type	35
wild type	polymorphic	140
wild type	wild type	72

A total of 132 seedlings were homoplastomic both in the *rpl23* gene and in the *rpl23* pseudogene (see Table 4). Most of these double homoplastomic seedlings (101 out of 132), carried the wild type sequence +++ in the *rpl23* gene, while 77 carried the **DEF** variant in the pseudogene. Moreover, 18 seedlings had the wild type sequence +++ in the *rpl23* pseudogene and five of these seedlings carried the variant **ABC** in the gene. Finally, 13 seedlings had sequences different to wild type both in the gene and in the pseudogene, *i*.*e*.: 12 seedlings carried variant ++**C** in the gene and variant **DE+** in the pseudogene; and one seedling had the combination of +**B+** in the gene and **D + F** in the pseudogene.

**Table 4:** Genetic variants of the *rpl23* gene and *rpl23* pseudogene in homoplastomic seedlings.

Number of seedlings	*rpl23* gene	*rpl23* pseudogene	Identical sequences
77	**+++**	**D E F**	yes
23	**+++**	**D E +**	no
12	**++C**	**D E +**	yes
11	**++C**	**+++**	no
5	**A B C**	**+++**	yes
2	**+B C**	**+++**	no
1	**+B+**	**D + F**	yes
1	**+++**	**D + F**	no

### *In silico* analysis for the presence of homologous sequences of the *rpl23* gene or the pseudogene amplicons in the nuclear and mitochondrial genomes of barley

We performed an *in silico* analysis to check for the presence of the complete *rpl23* gene and pseudogene amplicons in the barley genome. Both sequences were blasted against the *H*. *vulgare* mitochondrial and nuclear DNA. No hits were found when both amplicons were blasted to the mitochondrial genome of *H*. *vulgare* while the alignments showed that the amplicons would be contained completely in the nuclear genome. The *rpl23* amplicon matched against chromosomes 1, 2, 5, 6 and 7, and it was observed that some of the nuclear sequences were identical to the wild type plastome, while others were polymorphic. In total, 55 polymorphisms were identified and among them only two (G118A and A203G) corresponded to the five detected in *cpm* seedlings by CJE digestion and sequencing. In the nuclear sequences, those two polymorphisms were always accompanied by others, but none of them corresponded to those detected by CJE digestion and sequencing. Moreover, the only polymorphism found in the *rpl23* amplicon that located outside the gene region did not correspond to any of the 55 polymorphisms identified by blast in the nuclear genome.

The *rpl23* pseudogene amplicon matched against chromosome 6 and the nuclear sequence contained the five changes (T112G, A115G, A129T, InsG130, G199A) identified in the *rpl23* pseudogene of *cpm* seedlings.

## Discussion

### The high frequency of polymorphisms detected by CJE digestion in the *cpmrpl23* gene and/or pseudogene arose from illegitimate recombination of pre-existing differences in wild type barley between these two loci

A high proportion of *cpm* seedlings (92/304) were polymorphic for the *rpl23* gene (Table 1) and the proportion was even higher (197/304) for the *rpl23* pseudogene (Table 2). However, not a single polymorphism was detected among 40 wild type control seedlings, which included a wide range of barley entrances and also one *H*. *spontaneous*, a fact that strongly supports the influence of the *cpm* gene in the occurrence of these polymorphisms.

Given that by a blast analysis some of these polymorphisms were found in nuclear DNA sequences, it is appropriate to discuss if the polymorphisms detected by CJE digestions in *cpm* seedlings correspond to molecular differences that raised under the influence of the *cpm* genotype in the plastid *rpl23* gene and/or the pseudogene or, on the contrary, if we detected polymorphisms already existing in the nucleus. First of all, the sensitivity of detection of CJE/Cel I digestion of mismatches after heteroduplex formation in a mixture of different DNA molecules depends on the detection method. When using fluorescent dye detection (Li-cor), which is more sensitive than ethidium bromide, the pools of individuals are eightfold at most because higher dilutions of a polymorphism make it very hard to be detected by this technique. Furthermore, chloroplast genes are in much greater proportion than nuclear genes due to the high-copy number of plastids per cell. Genomic DNA extracts are naturally enriched for plastids and thus the plastome would be an easier target than low-copy nuclear genes for sequencing^17^ and amplification. In this sense, the identification of homoplastomic seedlings carrying polymorphisms in the *rpl23* gene and pseudogene is another situation difficult to explain under the hypothesis of the nuclear origin of the polymorphisms.

Regarding the blast analysis, among the 55 polymorphisms found in the nuclear DNA for the *rpl23* amplicon, only two of them were identified by CJE digestion and sequencing in *cpm* seedlings and in this sense, it is worth mentioning that one of them, G118A, was always found together with G115T, which was not found in the nuclear sequences. For the *rpl23* pseudogene amplicon, all the five polymorphisms were found together in chromosome 6, while when detected by CJE digestion and sequencing they showed with and without polymorphism G199A.

It is important to remark that all the polymorphisms observed by CJE digestions and sequencing corresponded to different combinations of the very same five polymorphisms already existing in wild type barley between the *rpl23* gene and pseudogene^18^. Besides, the five different polymorphisms located in similar positions in gene and pseudogene segregated as three blocks in correspondence with the distance between the different polymorphisms. The combination of the three blocks originated different genetic variants in both, gene and pseudogene. All these results strongly support the hypothesis that the polymorphisms in those regions, which were only detected in *cpm* seedlings, arose from increased illegitimate recombination of polymorphisms pre-existing in nature between the *rpl23* gene and pseudogene.

### Independent occurrence of polymorphisms in the *rpl23* gene or the pseudogene suggests that they originated in gene conversion

The analysis of both loci in the same seedling showed that the presence of polymorphisms in one of them was statistically independent from the presence of polymorphisms in the other. This result suggests that the polymorphisms more likely arose from gene conversion rather than from reciprocal exchanges, which would simultaneously affect both loci. In wheat and corn, Bowman *et al*.^15^ suggested that the sequence of the *rpl23* pseudogene undergoes a conversion process from DNA sequences of the *rpl23* gene, because of the lower divergence in pseudogenes in comparison to the surrounding non-coding regions. Subsequent work based on a phylogenetic analysis^16^ provided additional support to a model of gene conversion between the *rpl23* pseudogene and its functional counterpart in at least two lineages of the grass family. Our results support previous hypothesis and also show that gene conversion occurred in both directions, gene to pseudogene and *vice versa*.

### Higher diversity of genetics variants in the *rpl23* gene suggests that between the two copies of the gene there exist an additional recombination process apart from that recombining gene and pseudogene

The *rpl23* gene polymorphic seedlings carried six of the seven potential combinations of polymorphisms (Table 1), while the *rpl23* pseudogene polymorphic seedlings carried only three of the seven potential combinations (Table 2). Moreover, in the gene, 59 seedlings carried genetic variants that involve recombinations between block **B** and **C**, which are separated by 70 bp, while 22 seedlings carried genetic variants involving recombinations between blocks **A** and **B**, which are separated by only 14 bp. Our interpretation is that in *cpm* seedlings, recombination between the two copies of the gene occurs much more frequently than it does between the two copies of the gene and the pseudogene. Previous reports about homologous recombination of foreign DNA through gene conversion concluded that it is extremely rare in plastid sequences smaller than 50–100 bp^19,20^. However, it is worth to remark that recombination in the inverted repeats (IRs) have been several times proposed to occur not only by gene conversion. On the one hand, a copy correction mechanism like gene conversion between the IRs within a molecule is needed to maintain sequence identity between them. On the other hand, a high frequency of intramolecular recombination, probably crossing over between the IRs, was inferred in *Chlamydomonas*^21^, ferns^22^ and land plants^23–26^ from the fact that chloroplast genomes carrying two IRs, exist as an equimolar mixture of two isomers differing only in the relative orientation of their single-copy regions. On this basis, it can be hypothesized that the numerous recombinants involving small segments observed in the *rpl23* gene were a consequence of its location in the IRs. Whether the high recombination rates observed between the two copies of the gene have been increased under the influence of the *cpm* remains an open question.

Regarding the process of gene conversion between gene and pseudogene, it is reasonable to think that the genetic variants observed in the pseudogene would be more similar to those that originally arose from illegitimate recombination, than those observed in the gene, since the latter could be modified by selection pressure and/or additional recombination between the two copies of the gene. In such a case, we can speculate that probably gene conversion between gene and pseudogene frequently occurs by replacement of the entire DNA sequence in each locus and, recombinants between blocks separated by short segments would originate in the gene by crossing over between two copies of the gene, which are located in the IRs.

### A greater proportion of homoplastomic seedlings in the pseudogene in relation to the gene is in agreement with the idea regarding the higher rates of new allele fixation in the single copy regions compared to that in the IRs

As expected, homoplastomic seedlings for both gene and pseudogene were higher in Group A, which carried the *cpm* mutant during many more generations of self-pollination than Group B and therefore, mutants originated in early generations would have more opportunities of sorting out.

Besides, our results show that *cpm* seedlings polymorphic for the pseudogene (Table 2) had a greater proportion of homoplastomic seedlings than those polymorphic for the gene (Table 1), which is in agreement with the widely accepted idea that new alleles in the single copy regions have a higher rate of fixation than those in the IRs^27,28^. In this sense, the double copy number of genes located in the IRs compared to that of genes located in the single copy regions has been used to explain the lower frequency of mutations estimated for the former^29–33^.

### Several double homoplastomic seedlings show concerted evolution and lead to a dead end of potential variability arising from recombination between gene and pseudogene

The most common combination of double homoplastomic seedlings in both, gene and pseudogene, was +++ in the gene and **DEF** in the pseudogene (Table 4). As the sequences corresponding to blocks **D**, **E** and **F** in the pseudogene are identical to wild type sequences in the gene, both loci ended up having identical sequences. Therefore, such a genetic composition leads to a dead end in terms of potential variability through recombination between these loci. Moreover, if it is considered that sequences corresponding to blocks **A**, **B** and **C** in the gene are identical to wild type sequences in the pseudogene, the same situation occurs in other double homoplastomic seedlings *i*.*e*.:++**C** in the gene and **DE+** in the pseudogene; **ABC** in the gene and +++ in the pseudogene and +**B+** in the gene and **D + F** in the pseudogene (see Table 4). These findings can be considered an experimental verification of what has been defined as concerted evolution^34,35^.

### Relationship between block B (DelG133) and albinism suggests that some albino phenotypes could be attributed to the malfunction of the plastid ribosomes due to the lack of RpL23 protein

Several data support the hypothesis that some albino phenotypes are due to the detrimental effect of block **B** (Del133) in homoplastomic state. All 12 seedlings that carried block **B** (DelG133) in homoplastomy were albino and none of the seedlings carrying the wild type phenotype were homoplastomic for block **B**. Additionally, in *striata* seedlings carrying albino and normal green longitudinal stripes, block **B** in homoplastomic state was only observed in albino tissues. Moreover, albino seedlings homoplastomic for block **B** lacked the plastome encoded RbcL protein (Fig. 6), a fact that could be attributed to the malfunction of the plastid ribosomes due to the lack of RpL23 protein, whose essentiality was determined in tobacco even in heterotrophic conditions^36^. Similar observations have been made of an albino rice mutant having a substitution in the nuclear gene *prpl12*, which encodes the chloroplast ribosome protein PRPL12^37^ and the *albostrians* mutant of barley, which lacks plastid ribosomes and RbcL protein in albino tissues^38,39^.

### Illegitimate recombination between the *rpl23* gene and pseudogene is increased in *cpm* seedlings probably due to fails in the anti-recombination activity of a DNA mismatch repair (MMR) protein

The *cpm* seedlings investigated here were previously analyzed by a cpTILLING approach directed to a wide range of genes and intergenic regions, and those results support previous postulations regarding the involvement of the *Cpm* gene product in maintaining plastome DNA stability^13^. The spectrum of plastome polymorphisms detected in *cpm* seedlings mostly consisted of substitutions and small indels in microsatellites. Additionally, a peculiar pattern of polymorphisms observed in the *rpl23* gene and the presence of some big indels suggested that an increase of recombination rates also occurs in *cpm* seedlings^13^. In the present investigation, the hypothesis regarding the recombinational origin of *rpl23* gene polymorphisms in *cpm* seedlings was fully confirmed.

The spectrum of polymorphisms previously observed in *cpm* seedlings^13^ suggests the malfunctioning of a gene involved in the MMR system. The MMR system is critical for keeping mutation rates at low levels by correcting DNA replication errors. Besides, it contributes to genome stability by blocking recombination between divergent DNA sequences, such as the case of the *rpl23* gene and its pseudogene. Eukaryotic MMR proteins have been studied extensively in yeast and mammalian cells^40–42^, while knowledge about their role in maintaining plant genome stability, including plant genome organelles, is more scarce^43–46^.

### Conclusions and perspectives

It was confirmed that *rpl23* gene and *rpl23* pseudogene polymorphisms observed in *cpm* seedlings arose from increased rates of illegitimate recombination by gene conversion between these two loci. It is proposed that illegitimate recombination occurs in *cpm* seedlings as a consequence of failure of the MMR system anti-recombination activity that is usually in charge of preventing promiscuous recombination^42^. In the gene, the availability of polymorphisms acquired from the pseudogene allowed us to distinguish that between the two copies of the gene an additional recombination process occurs. This process probably includes crossing over, supporting previous postulations about the existence of reciprocal recombination between the IRs in land plants^24–26^. The higher proportion of homoplastomic seedlings in the pseudogene than in the gene agrees with the lower rate of new alleles fixation usually observed in genes located in the IRs^27,28^, and the genetic variants observed in double homoplastomic seedlings are a clear example of the consequences of concerted evolution^34,35^.

The overall landscape of polymorphisms previously observed in *cpm* seedlings also includes increased rates of substitutions and small indels in microsatellites, all supporting the postulation of the *Cpm* gene as a member of the DNA-MMR system^13^. The *cpm* mutant offers an interesting experimental material to investigate the mechanisms involved in maintaining the integrity of plant organellar DNA^47,48^ and their role in plant evolution^49^. Besides, as it was pointed out several times^3,4,8,50^, unstable genotypes like the *cpm* mutant can provide an alternative to the limited capacity of traditional techniques for inducing and isolating new plastome mutations.

## Materials and Methods

### Plant material

Most of the plant material consisted of DNA samples coming from 304 barley chloroplast mutator (*cpm*) seedlings^6^. This experimental material was previously used to conduct a plastome TILLING strategy for an extensive scanning of 33 plastome genes and a few intergenic regions^13^. These seedlings belong to six families maintained by natural self-pollination, which carried the *cpm* genotype through different numbers of generations. They were grouped into: Group A, consisting of two families that carried the homozygous mutator genotype (*cpm*/*cpm*) through 12 to 17 generations, and Group B consisting of four families that carried the mutator genotype for five generations^13^. In that investigation, 92 samples turned out to be polymorphic for the *rpl23* gene and they were used in the present work to further analyze the *rpl23* gene polymorphisms. In addition, all 304 seedlings mentioned above were tested in this work for *rpl23* pseudogene polymorphisms. Wild type seedlings belonging to two different control groups were also analyzed for polymorphisms in both the *rpl23* gene and the pseudogene. Group 1 consisted of 20 individual seedlings coming from different families derived from the very same parental genotype from which the *cpm* mutant was isolated after a mutagenic treatment^6^. Group 2 consisted of 20 individual seedlings representing a very wide range of barley accessions, which included malting and fodder commercial varieties and also some wild barley populations (Supplementary Table S1). In addition, we analyzed a few seedlings corresponding to progenies coming from *rpl23* polymorphic plants that segregated solid or *striata-albina* together with normal green seedlings.

All the seedlings were grown in a greenhouse for observation at the second leaf stage and for tissue extraction; later on they were transplanted to the field nursery and grown to maturity for observation and seed multiplication.

### DNA isolation

Genomic DNA was isolated from one or two leaves of individual seedlings using the micromethod described in Dellaporta^51^ with modifications. The tissue was ground with Dellaporta isolation buffer in the Fast Prep®-24 Instrument (MP Biomedicals, USA) and extracted with chloroform before DNA precipitation. DNA concentrations were measured using a spectrophotometer (Nanodrop, Thermo Scientific, Wilmington, DE, USA) and standardized to a concentration of 80 ng/µl.

### Analysis of the *rpl23* gene and *rpl23* pseudogene by celery juice extract (CJE) digestions

The 92 *cpm* DNA samples polymorphic for the *rpl23* gene were previously detected by amplification and celery juice extract (CJE) digestion of the *rpl2* amplicon (1,216 bp) obtained using the primers: rpl2F 5′- CACTTGCTGCCGTTACTCAA-3′ and rpl2R 5′-TCGAGGATCCAGAGAGGTGT-3′. The polymorphisms were determined by sequencing of the *rpl2* amplicon as described in Landau *et al*.^13^. In the present work, these 92 samples were re-amplified with the *rpl23* amplicon and then subjected to CJE digestion. The primers used for amplification of the *rpl23* amplicon (831 bp) were: rpl23F 5′-ATGGATGGAATCAAATACGCA-3′ and rpl2F 5′-CACTTGCTGCCGTTACTCAA-3′. The rpl23F primer is located at the beginning of the *rpl23* gene and in combination with the rpl2F primer amplifies both copies of the *rpl23* gene.

The screening for *rpl23* pseudogene polymorphisms was done by amplification and CJE digestion of the *rpl23* pseudogene amplicon (1,126 bp) in the 304 *cpm* seedlings. The primers used were: rpl23F 5′-ATGGATGGAATCAAATACGCA-3′ and hotspotR 5′-CATCCTCATGGCCTTTCTATCT-3′. The rpl23F primer is located at the beginning of the *rpl23* pseudogene.

The *rpl23* amplicon and the *rpl23* pseudogene amplicon were also used to identify polymorphisms by CJE digestion of DNA samples from wild type seedlings of the control groups.

In order to favor the heteroduplex formation during the screening assays for detection of *rpl23* gene and pseudogene polymorphisms, each sample was mixed with wild type DNA in a 1:1 ratio. The wild type DNA used for this purpose was the same used in our previous work^13^. In the analyses of the *rpl23* amplicon, the *cpm* DNA samples were also subjected to CJE digestion alone, without mixing with wild type DNA, to identify the homo- or hetero-plastomic state of the polymorphisms. The homo- or hetero-plastomic state was also estimated by the observation of one or two peaks in the electropherograms coming from the amplicon sequencing.

Primers were designed based on the chloroplast genome sequence of barley (*Hordeum vulgare*) [GenBank: NC_008590.1] using Primer3 software (v. 4.0.0, http://primer3.ut.ee/).

The PCR amplification conditions, slow re-annealing step, CJE digestion and polyacrylamide electrophoresis were performed according to Landau *et al*.^13^.

### Identification of polymorphisms in the *rpl23* gene and the *rpl23* pseudogene

The *rpl23* gene and the *rpl23* pseudogene amplicons showing digestion with CJE were analyzed by standard sequencing (Macrogen Inc, Korea) and the polymorphisms relative to the wild type sequences were determined.

Assembly and graphics were done with Vector NTI 10.0 Software (Thermo Fisher Scientific Inc, USA) and sequences were aligned with Clustal O (1.2.4). The barley plastome figure was done with OGDraw^52^ online software.

### Protein extraction and Western blot

The soluble protein fraction of the thylakoid isolation protocol was obtained according to Guiamét *et al*.^53^. The concentration of soluble proteins was assessed by micro BCA protein assay kit (Thermo Fisher Scientific Inc, USA). Soluble fraction proteins were separated by SDS-PAGE and immunoblotted with rabbit anti Rubisco large subunit (RbcL) antiserum (Agrisera, Sweden) and rabbit *E*. *coli* RecA antiserum (Abcam, UK) to determine the presence of the chloroplast-encoded subunit of the Rubisco (RbcL) and the chloroplast-targeted ortholog of the *E*.*coli* RecA protein (cpRecA). Twenty micrograms of soluble proteins were electrophoretically separated in 13% (w/v) SDS-polyacrylamide gels, transferred to nitrocellulose membranes and probed with rabbit antibodies against RbcL and cpRecA proteins. The immunodetection was performed with SuperSignal West Dura extended duration substrate (Thermo Fisher Scientific Inc, USA) according to the manufacturer’s description.

### *In silico* analyses for the presence of homologous sequences of the *rpl23* gene and pseudogene amplicons in the nuclear and mitochondrial genome of barley

The presence of the *rpl23* gene and pseudogene amplicons in the nuclear and mitochondrial genomes of barley was investigated by Blastn. The sequences of both amplicons were blasted against each of the seven chromosomes of *Hordeum vulgare sub*. *vulgare cv*. *Morex* from *EnsemblPlants* (ftp://ftp.ensemblgenomes.org/pub/release-43/plants/fasta/hordeum_vulgare/dna/) and IPK barley blast server (https://webblast.ipk-gatersleben.de/barley_ibsc/) using barley pseudomolecules masked apr2016 as database. For the mitochondrial genome, the Genbank sequence of *Hordeum vulgare sub*. *vulgare cv*. *Haruna Nijo* (https://www.ncbi.nlm.nih.gov/nuccore/AP017301) was used.

## Supplementary information

Supplementary information

## Data Availability

All data generated or analyzed during this study are included in this published article (and its Supplementary Information Files).
