# Supplementary material for: The rpl23 gene and pseudogene are hotspots of illegitimate recombination in barley chloroplast mutator seedlings
Source: Sci Rep. 2019 Jul 10;9:9960. doi: 10.1038/s41598-019-46321-6 (PMC6620283; doi:10.1038/s41598-019-46321-6)
Supplement: Supplementary file 1 — Supplementary information [file 41598_2019_46321_MOESM1_ESM.pdf]

The *rpl23* gene and pseudogene are hotspots of illegitimate recombination in barley chloroplast mutator seedlings

Lencina F[1], Landau AM[1], Petterson ME[1], Pacheco MG[1], Kobayashi K[2], Prina AR[1*]

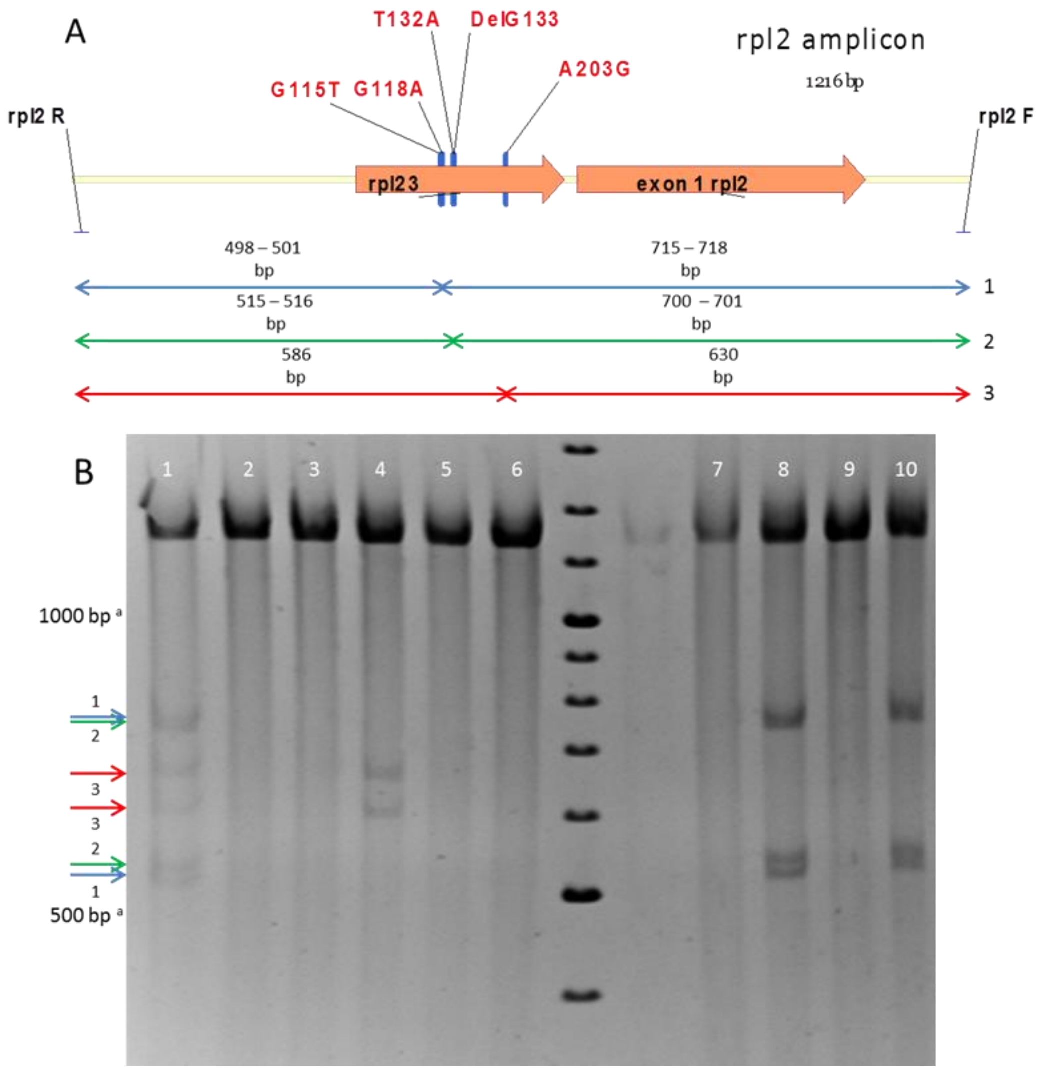

Supplementary Figure S1. A The *rpl2* amplicon and the polymorphisms detected in the *rpl23* gene. **B** CJE digestions of *rpl2* amplicons on a nondenaturing 3% polyacrylamide gel. Different *cpm* seedlings show different digestion patterns because they had different combinations of polymorphisms: seedling 1 carries G115T, G118A, T132A, DelG133 and A203G; seedlings 2, 3, 5, 6, 7 and 9 do not carry any polymorphism; seedling 4 carries A203G; seedlings 8 and 10 carry G115T, G118A, T132A and DelG133. [a]The samples run slower than the molecular weight marker probably due to the components of the CJE digestion mixture.

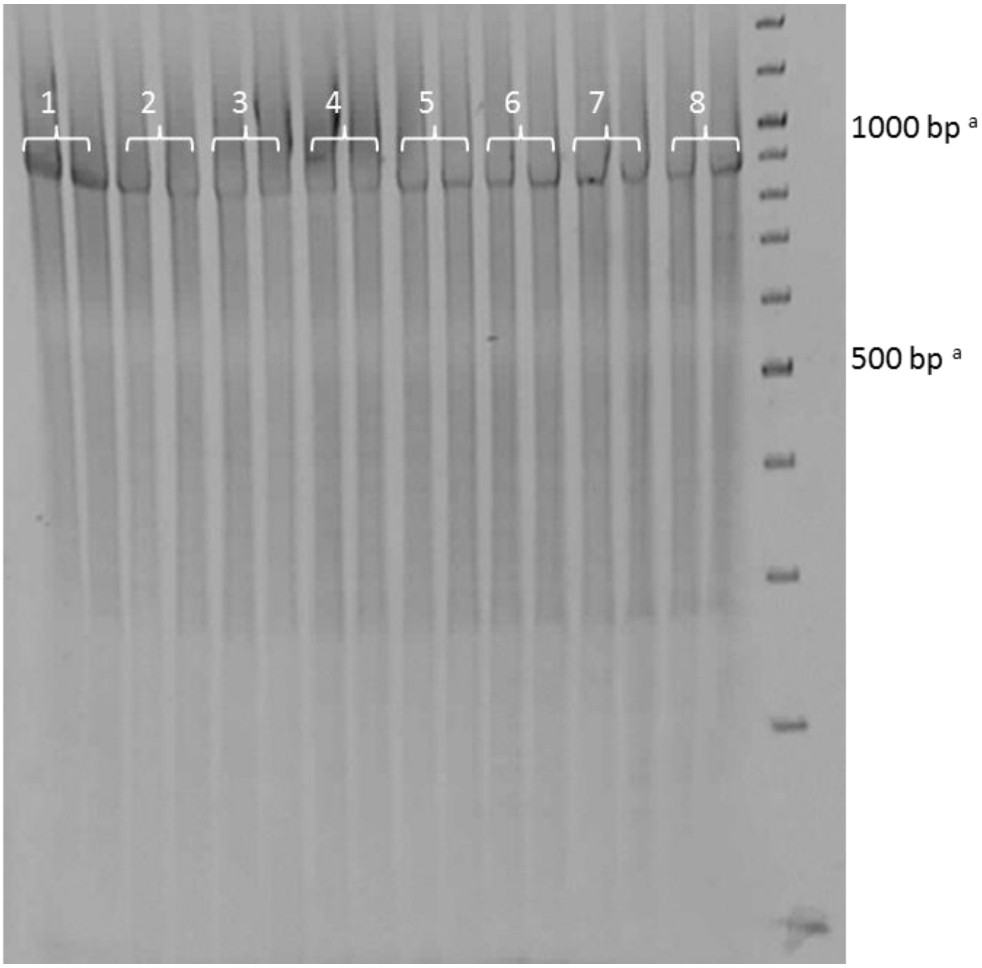

Supplementary Figure S2. CJE digestions of the *rpl23* amplicons on a nondenaturing 3% polyacrylamide gel of control seedlings. The first and second lanes of each sample correspond to the digestion of the sample alone and mixed with wild-type DNA in a 1:1 ratio, respectively. [a]The samples run slower than the molecular weight marker probably due to the components of the CJE digestion mixture.

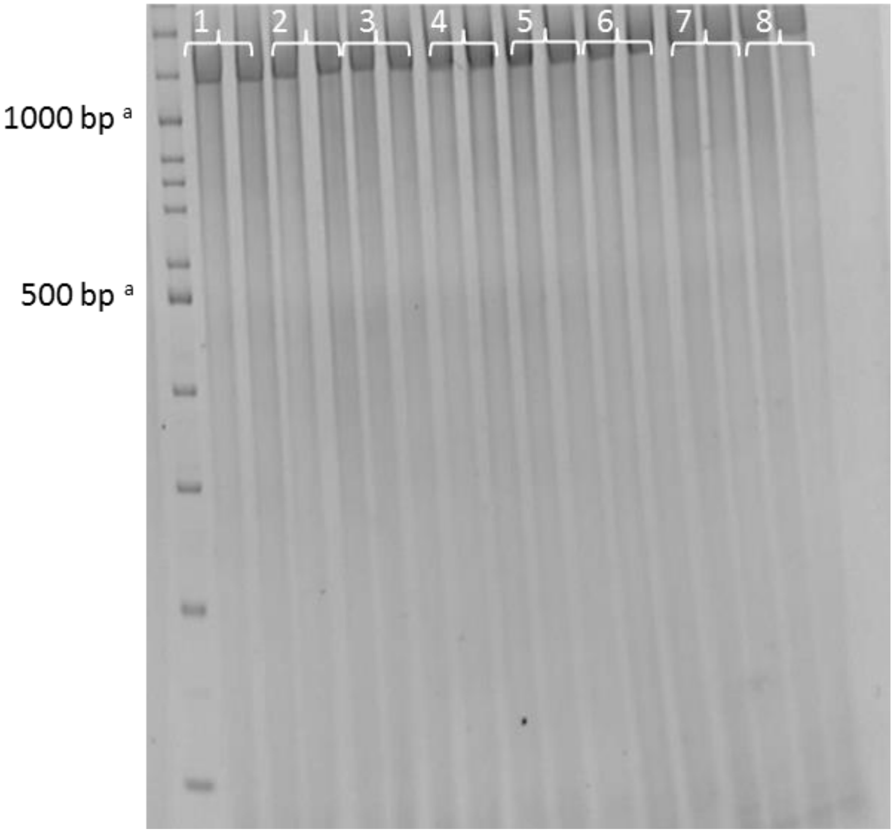

Supplementary Figure S3. CJE digestions of the *rpl23* pseudogene amplicons on a nondenaturing 3% polyacrylamide gel of control seedlings. The first and second lanes of each sample correspond to the digestion of the sample alone and mixed with wild-type DNA in a 1:1 ratio, respectively. [a]The samples run slower than the molecular weight marker probably due to the components of the CJE digestion mixture.

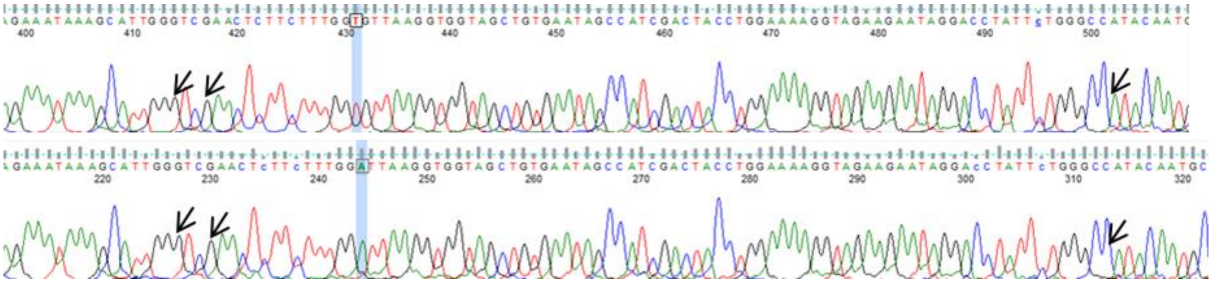

Supplementary Figure S4. Electropherograms of the *rpl23* gene amplicon of a wild type seedling (upper) and a *cpm* seedling (lower) carrying the genetic variant +B+ (T132A and DelG133) in homoplastomy. The position of the block B is highlighted in blue and the arrows indicate the positions where blocks A and C should be located if they were present.

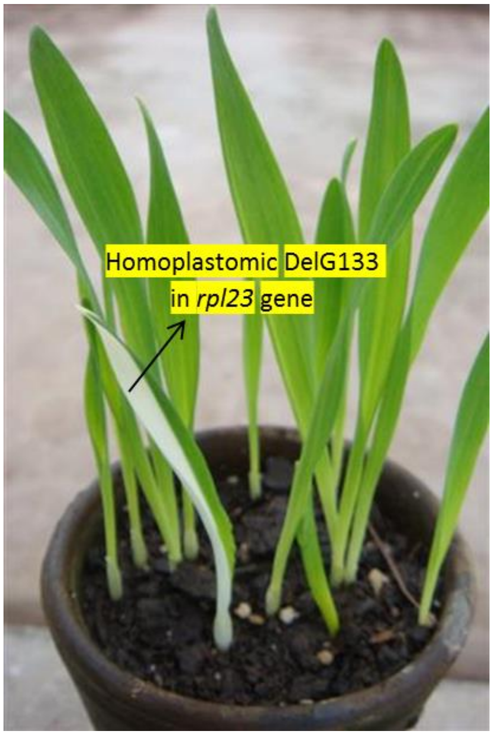

Supplementary Figure S5. A s*triata* seedling carrying DelG133 in the *rpl23* gene in the *albino* tissue.

```
WT            MDGIKYAVFTEKSLRLLGKNQYTFNVESGFTKTEIKHWVELFFGVKVVAVNSHRLPGKGR  60
truncated     MDGIKYAVFTEKSLRLLGKNQYTFNVESGFTKTEIKHWFKLFFGLRW-------------  47
              **************************************.:****::

WT            RIGPILGHTMHYRRMIITLQPGYSIPLLDREKN   93
truncated     ---------------------------------   47
```

Supplementary Figure S6. Alignment of the truncated and wild type Rpl23 protein sequences.

Hordeum vulgare accessions	IGEAF Code number	BR Code[d]
Engledow India[a]	08-1001	ENG 3
Cebada Negra[a]	08-1002	
Montecristo[a]	08-1003	
Gopal[a]	08-1004	GOP
Nigrate[a]	08-1005	NIG
Chinerme[a]	08-1006	CHIA
Grandpa[a]	08-1007	
Colses IV[a]	08-1010	
Colses V[a]	08-1011	
Uzu[a]	08-1012	
Abacus[a]	08-1014	ABA 1
Trebi[a]	08-1034	TRE 4
Malteria Heda[c]	08-1052	
Lemna Naranja[c]	10-1013	
Scarlet[b]	10-1434	
Golden Promise[a]	10-1435	GOLD 5
Q. Ayelen[b]	12-1212	
Q. Paine[b]	12-1213	
Andreia[b]	12-1214	

Hordeum spontaneum accessions	IGEAF Code number
CI 4140[a]	08-1009

Supplementary Table S1. Barley genotypes of the Group 2 control population. [a]Experimental material introduced at IGEAF. [b]Old and present commercial varieties cultivated in Argentina. [c]Mutant isolated at the IGEAF. [d]Barley Register Code (Baun, B., Thompson, B.K., Grant Bailey, L. & Brown, M. *Barley Register (A First Reporter) Research Branch* (Agriculture Canada, 1981).

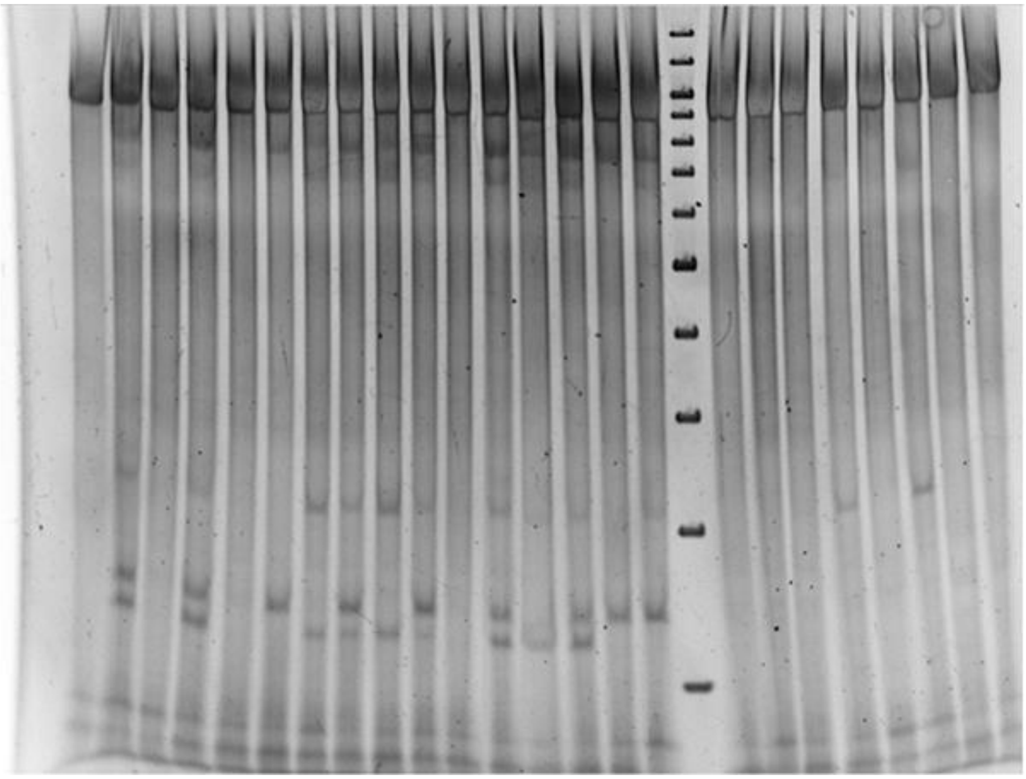

Fig. 2B Full-length gel

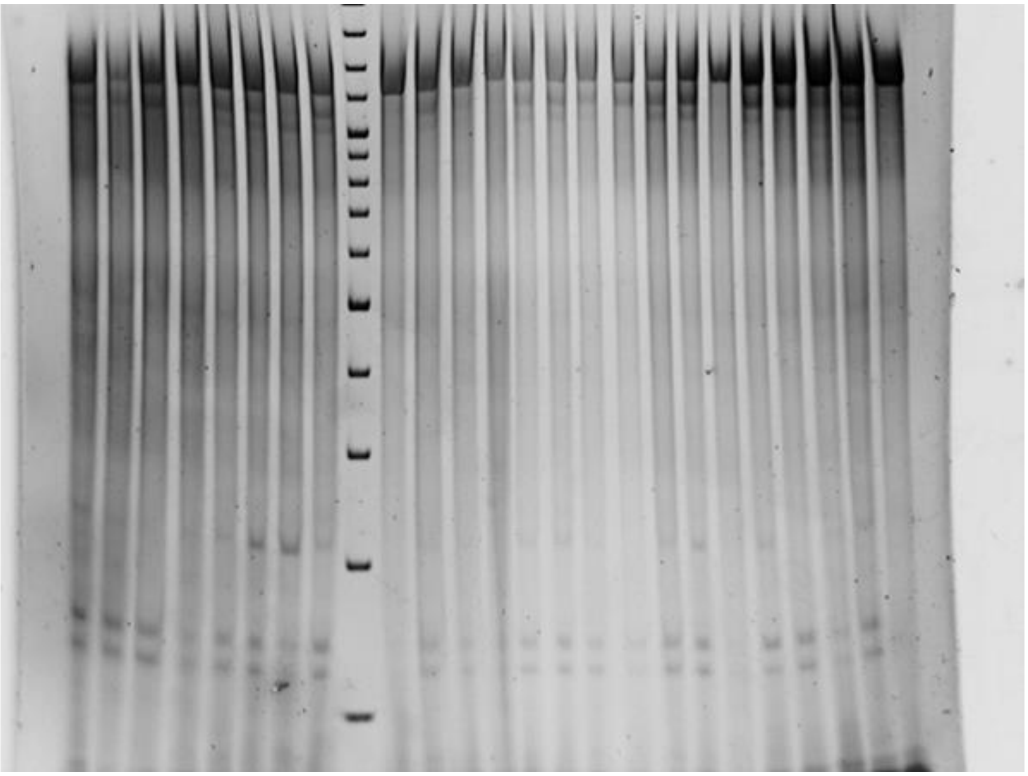

Fig. 4B Full-length gel

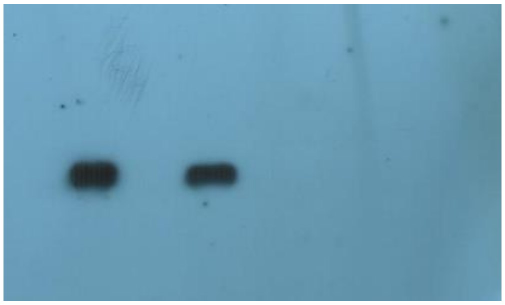

Fig. 6 Full-length upper blot

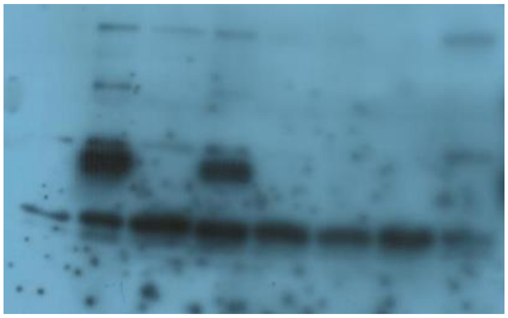

Fig. 6 Full-length lower blot
